# A durable hydrophobic photothermal membrane based on a honeycomb structure MXene for stable and efficient solar desalination†

**DOI:** 10.1039/d3ra08157e

**Published:** 2024-04-02

**Authors:** Chunjiao Liu, Peng Wu

**Affiliations:** a Xinyang Vocational and Technical College Xinyang Henan 464000 China liuchunjiao_2006@126.com

## Abstract

Solar powered water evaporation is a green and environmentally friendly water treatment technology, which is a hot research topic for water purification at present. Advanced structural design and hydrophilic photothermal materials have achieved efficient solar evaporation of pure water, but the long-term stability of high salinity desalination has become a problem that cannot be ignored in practical applications. In order to solve this problem, a hydrophobic honeycomb structure MXene/AuNFs composite membrane was proposed in this paper, which used the three-dimensional highly porous microstructure of MXene and multibranched structure of gold nanoflowers particles to improve the light absorption and photothermal conversion efficiency of MXene/AuNFs. At the same time, the surface of the composite membrane was modified with hydrophobic fluorosilane 1*H*,1*H*,2*H*,2*H*-perfluorodecyltriethoxysilane (PFTE). The hydrophobic layer can prevent the accumulation of salt particles on the surface of the membrane, so that the composite film can continue to produce water vapor in a high salt environment. With high utilization rate of light energy, multiple-level geometrical structures of MXene for rapid water transport on the filter membrane and salt barrier on the membrane good stability, the hydrophobic MXene/AuNFs achieves solar evaporation rate of 1.59 kg m^−2^ h^−1^ and solar conversion efficiency is 97.8%, and stable operation under simulated sea water conditions under one sun irradiation over more than 10 cycles. The hydrophobic MXene/AuNFs membrane proved to be an efficient and stable photothermal material for solar desalination.

## Abstract 2

A biomimetic honeycomb structure and the modification of AuNFs were used to improve the photothermal conversion capability of MXene. Efficient solar driven water evaporation is achieved by covering the MXene/AuNFs with a hydrophobic layer.

## Graphical abstract

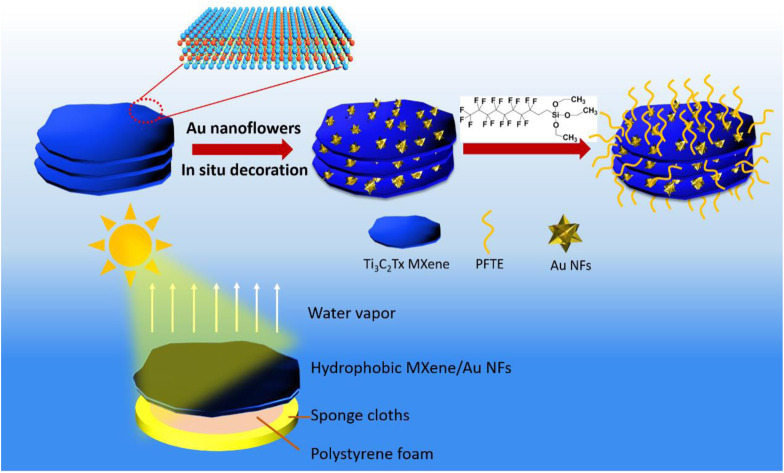

## Introduction

With the increasing shortage of available drinking water resources,^1,2^ how to use green technology to obtain more clean water production is a key issue at present. As a sustainable and renewable energy, solar energy has been developed and utilized in many fields, including solar cells,^3–7^ photocatalysis^8–11^ and photothermal conversion.^12–14^ In particular, photothermal conversion has been widely studied in recent years in photothermal therapy, photothermal water evaporation, and photothermal braking. Solar steam generation is an economical and sustainable technique for extracting fresh water from seawater desalination and purification. Unlike for traditional reverse osmosis desalination systems, solar desalination energy consumption comes from unlimited solar energy, saving energy and higher resource utilization. In order to achieve high water evaporation rates and light-to-heat conversion efficiency, the primary focus is to design photothermal material with a wide range of absorptivity, low thermal conductivity, sustainability, and high solar steam generation efficiency. Currently, the popular photothermal materials with efficient light-to-heat conversion include carbon materials,^15–17^ metallic nanoparticles,^18–20^ semiconductors,^21,22^ which have been widely used in solar powered water evaporation technology. For example, Ibrahim *et al.* reported a carbonaceous sawdust/ES-PANI composite decorated with silver sulfide, which enabled a clear salt-rejection capability.^23^ Chen and co-workers employed the tetrapyridylporphyrin as an interface in water evaporation with a water evaporation rate of 0.69 kg m^−2^ h^−1^ and a stable voltage up to 60 mV.^24^ Furthermore, laser-treated wood and carbonized pine cone were chosen as photothermal materials due to their high conversion efficiency and evaporation flux reported by Wang *et al.*^25^ and Goharshadi *et al.*^26^ Although diverse approaches have been employed to improve the light-to-heat conversion efficiency, in practice, the overall steam production capability and water production capacity still have a lot of room for improvement. In typical solar thermal evaporation systems often have salt crystals on the surface of the photothermal film, which leads to a significant reduction in the efficiency of steam generation and the destruction of the film.^27,28^ Hydrophobic surfaces naturally repel water, which can prevent salt precipitation during solar desalination.^29^ As a result, to design a hydrophobic photothermal membrane with high light-to-heat conversion efficiency becomes a key scientific challenge for long-term stable solar desalination.

Ti_3_C_2_T_*X*_ MXene, a new type of 2D layer material, have attracted great attention due to its unique properties since they were first reported in 2011 by Gogotsi, Barsoum, and colleague.^30,31^ It has been demonstrated that Ti_3_C_2_T_*X*_ MXene has excellent light absorption from UV to NIR,^32^ and the internal photothermal conversion efficiency of Ti_3_C_2_T_*X*_ MXene was reported to be 100%,^33^ suggesting Ti_3_C_2_T_*X*_ MXene is a perfect photothermal material and has been widely used in many fields, such as photothermal therapy,^34,35^ solar water desalination^36,37^ and optical devices^38,39^*etc.* Moreover, compared to other 2D nanomaterials, Ti_3_C_2_T_*X*_ MXene has easily controlled surface functional groups that can modulate the properties of Ti_3_C_2_T_*X*_ MXene for various missions.^40,41^ MXenes' properties can be adjusted by selecting combinations of transition metals, X elements, and noble metals. Alternatively, gold nanoparticles and nanocomposites, as photothermal agents with unique surface plasmon resonance (SPR) properties, show important applications in anticancer related detection. We have previously reported an Au NP@MXene membrane with spherical gold nanoparticles as a highly efficient high-temperature pulse generator for laser ignition of energetic materials.^42^ While the solar steam generation efficiency of Au NP@MXene composite membrane is not prominent, thus limiting its application. Gold nanoflowers (AuNFs) are gold nanoparticles with flower-like branching structures. Compared with 2D structures, multi-branched AuNFs have larger surface area, higher reactivity and strong localized surface plasmon resonance (LSPR) effect.^43–45^ It is expected that the combination of Ti_3_C_2_T_*X*_ MXene-based composites with AuNFs could be especially advantageous in solar seawater desalination applications, owing to wide spectrum absorption capability and high photothermal conversion efficiency for solar steam generation.

**Fig. 1:**
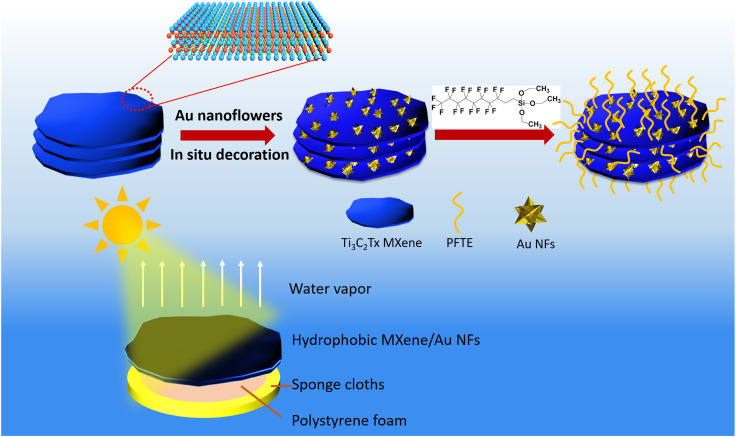
A schematic describing the fabrication of hydrophobic MXene/AuNFs composite membrane and the mechanism of generating HTP under laser irradiance.

In this work, we report the first example of constructing of honeycomb structure hydrophobic membrane based on a Ti_3_C_2_T_*X*_ MXene film assembled with plasmonic AuNFs as an effective light absorber for solar driven evaporation. The hydrophobic surface of MXene/AuNFs composite membrane modified with 1*H*,1*H*,2*H*,2*H*-perfluorodecyltriethoxysilane (PFTE) which can inhibit salt-blocking and increase water evaporation rate (Fig. 1). The honeycomb structure can be used as a transport channel for water and an escape channel for water vapor. The 3D AuNFs dramatically enhanced the light absorption capacity of Ti_3_C_2_T_*X*_ MXene in ultraviolet, visible and near-infrared regions. The resultant hydrophobic MXene/AuNFs with an underlying thermal barrier show a high conversion of light to water evaporation efficiency up to 97.8% under 1 sun light illumination (1 kW m^−2^), which is comparable to other solar steam generator systems currently published. Furthermore, the hydrophobic MXene/AuNFs interfacial water evaporator may be a viable large-scale used in many fields such as seawater desalination and sewage treatment based on its simple fabrication approach and high evaporation rate. This work proves that hydrophobic MXene/AuNFs nanocomposite is a promising photothermal material for solar-driven steam generation and provides a concept of nanoarchitectonics may solve the key problem of dissatisfactory photothermal efficacy for practical solar energy utilization.

## Experimental section

### Materials

Chloroauric acid (HAuCl_4_·4H_2_O), lithium fluoride (LiF), hydrochloric acid (HCl, ACS reagent, 37% w/w) and sodium citrate were all purchased from Macklin Biochemical Co., Ltd (Shanghai, China). Titanium aluminum carbide powder (Ti_3_AlC_2_, 400 mesh) was acquired from Beike 2D materials Co., Ltd (Beijing, China). 1*H*,1*H*,2*H*,2*H*-perfluorodecyltriethoxysilane (PFTE, 98.00%) was purchased from Energy Chemical.

### Synthesis of Ti_3_C_2_T_*X*_ MXene nanosheets

The few-layer Ti_3_C_2_T_*X*_ dispersion was synthesized by etching the Ti_3_AlC_2_ MAX phase with lithium fluoride (LiF)-hydrochloric acid (HCl) strong acid system. The concentration of HCl solution used is 9 M. Firstly, a 1 g amount of LiF was added into 20 mL of the HCl solution and sonicated for 30 min. Then, 2 g of Ti_3_AlC_2_ was gradually added into the mixed solution at room temperature with magnetic stirring for 24 h. The obtained acidic mixture was repeatedly washed with deionized H_2_O *via* centrifugation at 3500 rpm several times until the pH of the supernatant was >6. The delamination of Ti_3_C_2_T_*X*_ was conducted by dispersing the clay-like Ti_3_C_2_T_*X*_ in ethanol and ultra-sonicated for 2.0 hours. The Ti_3_C_2_T_*X*_ suspension was centrifuged at 10 000 rpm and dispersed in deionized water. A stable colloid suspension of few-layered Ti_3_C_2_T_*X*_ nanosheets was collected by sonication treatment followed by 1 h of centrifugation at 3500 rpm of delamination of Ti_3_C_2_T_*X*_.

### Preparation of hydrophobic MXene/AuNFs membrane

200 mL 0.01% HAuCl_4_·4H_2_O was continuously stirred and heated, then quickly add 3 mL 1% sodium citrate and keep the mixture boiling for 20 minutes. The obtained gold seed solution was cooled to room temperature and then stored at 4 °C.

After adjusted the pH of the 200 mL of 0.01% HAuCl_4_·4H_2_O to 11.5 with 1 M NaOH, 5 mL Ti_3_C_2_T_*X*_ MXene (5 mg mL^−1^), 1.2 mL of 0.02 m hydroxylamine hydrochloride was mixed with 48 mL of the prepared gold seed solution under strong stirring a few minutes and overnight. The mixture was separated by vacuum filtration and then the MXene/AuNFs film was obtained by freeze-drying. According to the above method, we prepared MXene/AuNFs membrane with various AuNFs content. The weight percentage of AuNFs in MXene/AuNFs composite was set at 17, 32 and 39 wt%.

100 mg of 1*H*,1*H*,2*H*,2*H*-perfluorodecyltriethoxysilane (PFTE) was added to absolute ethanol to make a 5 mg mL^−1^ solution. Then, the above-mentioned ethanol solution of PFTE were added into 2 mL of MXene/AuNFs solution and stir for 2 hours at room temperature. The hydrophobic MXene/AuNFs membranes were finally obtained in the same way as MXene/AuNFs. The preparation method of MXene membrane is the same as that of hydrophobic MXene/AuNFs membrane. First, 2 mL of MXene dispersion is separated from solid and liquid, and then the solid is freeze-dried to obtain MXene membrane. The thickness of the prepared hydrophobic MXene/AuNFs photothermal film is ∼18 μm (Fig. S1†).

### Material characterization

The surface microstructure of the Ti_3_C_2_T_*X*_ MXene based samples were characterised by transmission electron microscopy (TEM, Tecnai F30, FEI, Eindhoven, The Netherlands) and scanning electron microscopy (SEM, Ultra 55, Carl Zeiss, Germany). The thickness of MXene and hydrophobic MXene/AuNFs layer was obtained by atomic force microscopy (AFM, Dimension ICON, Bruker AXS, Germany). The atomic ratios of fluorine to titanium of MXene/AuNFs and hydrophobic MXene/AuNFs films were characterized using the X-ray photoelectron spectroscopy (XPS) (PHI 5000 VersaProbe). X-ray diffraction (Bruker D8 Advance, Germany) was performed to characterize the crystal structure of Ti_3_C_2_T_*X*_ MXene and TiO_2_ nanoparticles. The zeta potential values of solutions were estimated using a Zetasizer Nano ZS90 (Malvern Instrument, UK). The water contact angle (CA) of all the membranes were performed on a CA measurement system (Kruss DSA100, Germany). The absorption performance of MXene and MXene/AuNFs were measured from 200 to 1100 nm wavelength using a UV-visible spectroscopy (UV-8000S, METASH, Shanghai, China). Zeta potential was measured by a Malvern Zetasizer Nano ZS90 (Malvern Panalytical, UK). Fourier transform infrared (FT-IR) spectra were measured with the Nicolet iS10 spectrometer (Thermo, America) in the range of 400 to 4000 cm^−1^.

### Steam generation experiment

To improve the water evaporation efficiency of photothermal membranes, a homemade solar evaporation device is developed with polystyrene (PS) foam and cellulose sponge acting as thermal insulating layer and water transfer channels shown in Fig. 1. Solar-powered water evaporation experiments were performed in the laboratory, where the ambient temperature and the relative humidity during tests was about ∼22 °C and ∼26% with a solar simulator (CEAULIOHT Ltd Co., CEL-S500, China). The weight changes were recorded by electronic analytical scale (Mettler-Toledo, ML204). The surface temperature of MXene, MXene/AuNFs and hydrophobic MXene/AuNFs membranes were measured using an IR camera (FLIR ONE Pro).

## Results and discussion

### Structures and properties of hydrophobic MXene/AuNFs composite

**Fig. 2:**
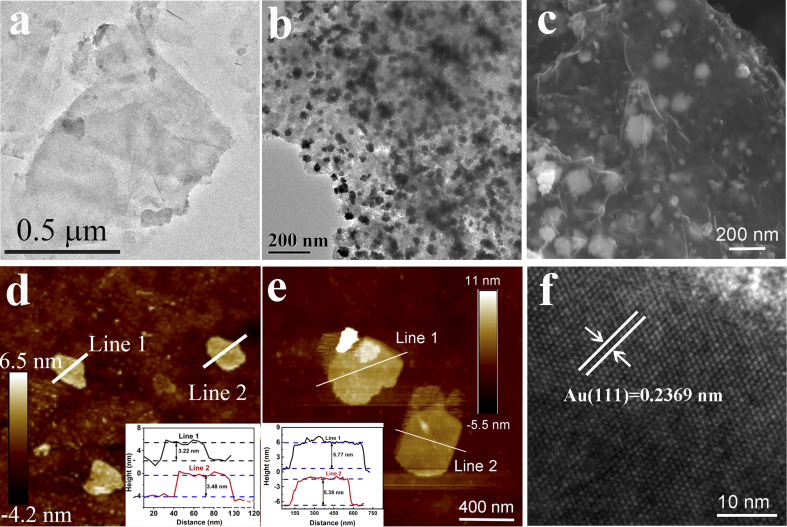
Low-resolution TEM of (a) Ti_3_C_2_T_*X*_ MXene and (b and c) hydrophobic MXene/AuNFs nanosheets. AFM images of (d) the Ti_3_C_2_T_*X*_ MXene nanosheets and (e) hydrophobic MXene/AuNFs composite. (f) High-resolution TEM image of the hydrophobic MXene/AuNFs nanosheets.

**Fig. 3:**
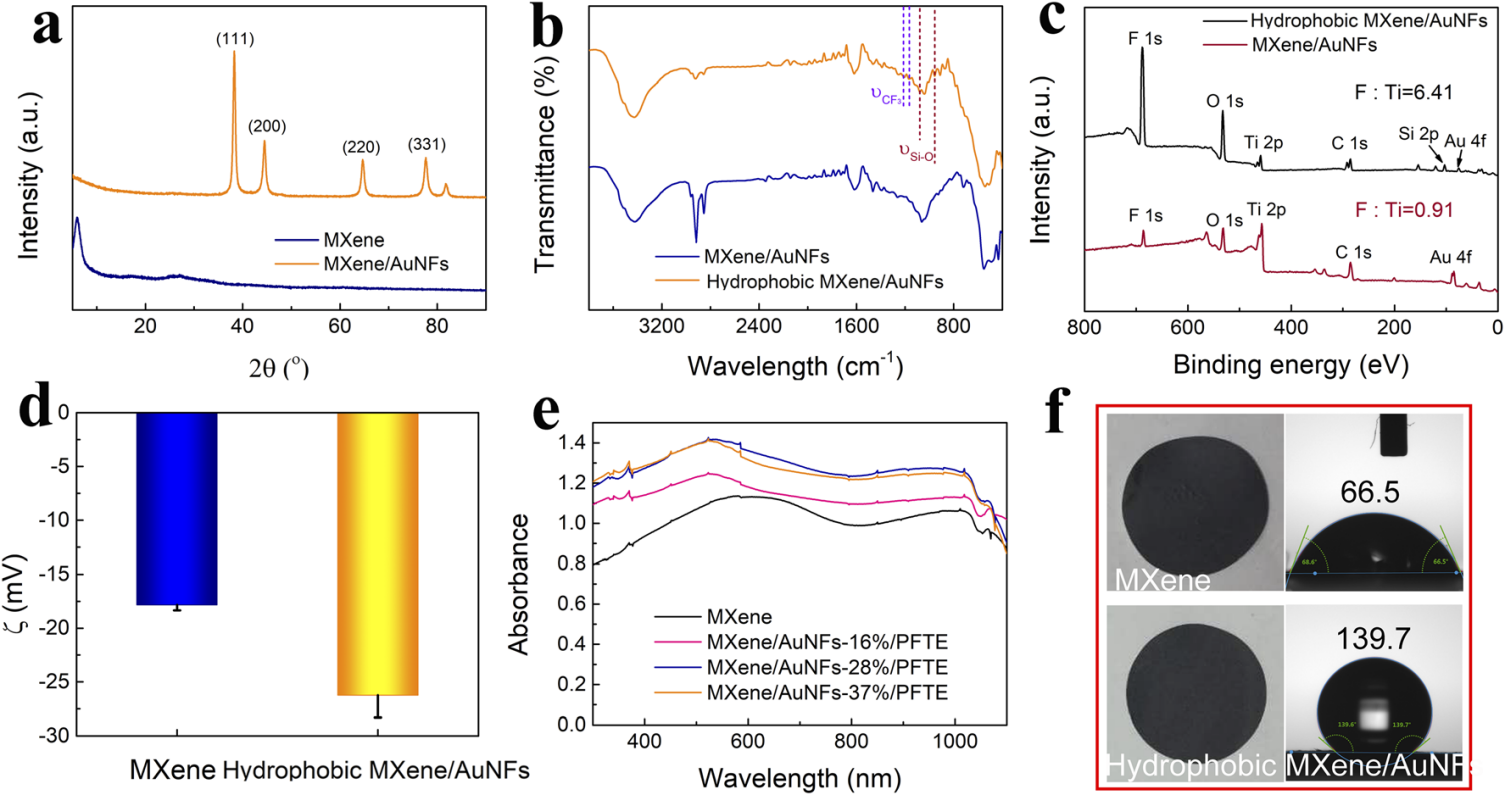
(a) XRD patterns of the Ti_3_C_2_T_*X*_ MXene nanosheets and MXene/AuNFs nanocomposites. (b and c) FTIR spectra, XPS survey spectra of MXene/AuNFs and hydrophobic MXene/AuNFs nanocomposites. (d) Zeta potentials of MXene and hydrophobic MXene/AuNFs. (e) UV-vis-NIR absorption spectrum of Ti_3_C_2_T_*X*_ MXene and hydrophobic MXene/AuNFs membrane with different AuNFs loadings. (f) The optical photograph and contact angle of MXene and hydrophobic MXene/AuNFs membranes.

Colloidal Ti_3_C_2_T_*X*_ MXene suspensions were synthesized using a chemical exfoliation method according to the previous literature.^46,47^ As shown in Fig. 2a and d, the transmission electron microscopy (TEM) and atomic force microscopy (AFM) images prove the formation of the Ti_3_C_2_T_*X*_ MXene nanosheets with a size of ∼0.5 μm and thickness of 3 nm. A variety of characterization techniques were used to elucidate the micro-structure of the hydrophobic MXene/AuNFs composite. The TEM and SEM images of the hydrophobic MXene/AuNFs hybrid in Fig. 2b and c illustrate that the AuNFs are uniformly dispersed on the surface of MXene nanosheets and showed complicated flower-like structure. In addition, AFM confirmed the thickness of hydrophobic MXene/AuNFs hybrid nanosheets in Fig. 2e that the typical nanosheets have thicknesses of ∼5.77 nm and ∼5.38 nm, while pure MXene nanosheets were about 3 nm (Fig. 2d), indicating that after loading of gold nanoparticles and hydrophobic treatment, the MXene layer was thickened. The gold nanoparticles and PFTE were successfully connected to the MXene surface. The roughness measured by AFM also shows that the roughness of treated MXene (Ra: 1.45 nm) increases significantly compared with that of pure MXene (Ra: 1.27 nm). The high-resolution TEM image demonstrated that AuNFs were single crystals. Fig. 2f shows the interplanar spacing of lattice with *d* (111) of ∼0.2369 nm. The XRD pattern (Fig. 3a) shows the characteristic peaks of AuNFs corresponding to the (111), (200), (220), and (311) planes. The above characterization results confirmed the successful attachment of AuNFs to the surface of MXene nanosheets. The compositions of MXene/AuNFs and PFTE-treated MXene/AuNFs films were further analyzed *via* Fourier transform infrared spectroscopy (FTIR) and X-ray photoelectron spectroscopy (XPS) to illustrate the presence of the fluoride alkyl silane structures on the surface of MXene/AuNFs, as shown in Fig. 3b and c. For the PFTE-treated MXene/AuNFs films, the typical peaks for the CF_3_ stretching vibrations (*υ*_(CF_3_)_) at 1212 and 1116 cm^−1^, while absorption band at 1078 and 961 cm^−1^ can be assigned to the stretching vibrations of Si–O, the above data are consistent with those previously reported in the literature^48^ and indicate that the possible bonding interaction between PFTE and MXene/AuNFs. As shown in Fig. 3c, the chemical composition of MXene/AuNFs and hydrophobic MXene/AuNFs was conducted by XPS. The F/Ti atomic ratio of hydrophobic MXene/AuNFs (6.21) was larger than the MXene/AuNFs (1.07), which indicated that much fluorinated functional groups were connected to the MXene, re-confirmed the efficient surface modification of MXene/AuNFs with PFTE. The zeta potentials of MXene/AuNFs and hydrophobic MXene/AuNFs were measured and presented in Fig. 3d. Obviously, the mean zeta potential of hydrophobic MXene/AuNFs (−26 mV) decreased significantly compared with that of MXene/AuNFs (−18 mV), indicating the assembly of the PFTE on the surface of MXene/AuNFs, which is consistent with above results.

Efficient solar-driven water evaporation depends on the efficiency of light absorption on the device surface, so materials with efficient light absorption are preferable. The UV-vis spectrum (300–1100 nm) for MXene and the composite membranes loading AuNFs with different mass ratios was recorded and presented in Fig. 3e. As expected, a significant enhancement in the overall light absorption of the hydrophobic MXene/AuNFs hybrid membrane was achieved with AuNFs loadings and reached a maximum at 28 wt%. After MXene loaded with AuNFs and PFTE, its surface roughness increases significantly, as can be seen in Fig. S2.† The multiscale surface texturing of the MXene based composite membrane induced by AuNFs and PFTE addition may cause considerable deviations in the scattering and reflection of light,^49,50^ leading to higher absorption of the solar than that of a flat smooth surface, and thus a higher photothermal conversion efficiency.

As can be seen in the UV-vis spectrum, the unique absorption peak of MXene at 600 nm has a blue-shift with the addition of AuNFs, which indicates that there is an interaction between MXene and adjacent AuNFs. In the subsequent experiments, the loading of AuNFs in hydrophobic MXene/AuNFs hybrid membrane for all the tests were 28 wt%. The wetting properties directly affects the light-water vapor conversion efficiency of photothermal materials. The contact angle test was conducted as shown in Fig. 3f. Owing to the chemical composition with PFTE, the wetting properties of MXene changed from being hydrophilic (CA: 66.5°) to superhydrophobic (CA: 139.7°).

### The steam generation experiment of hydrophobic MXene/AuNFs film

**Fig. 4:**
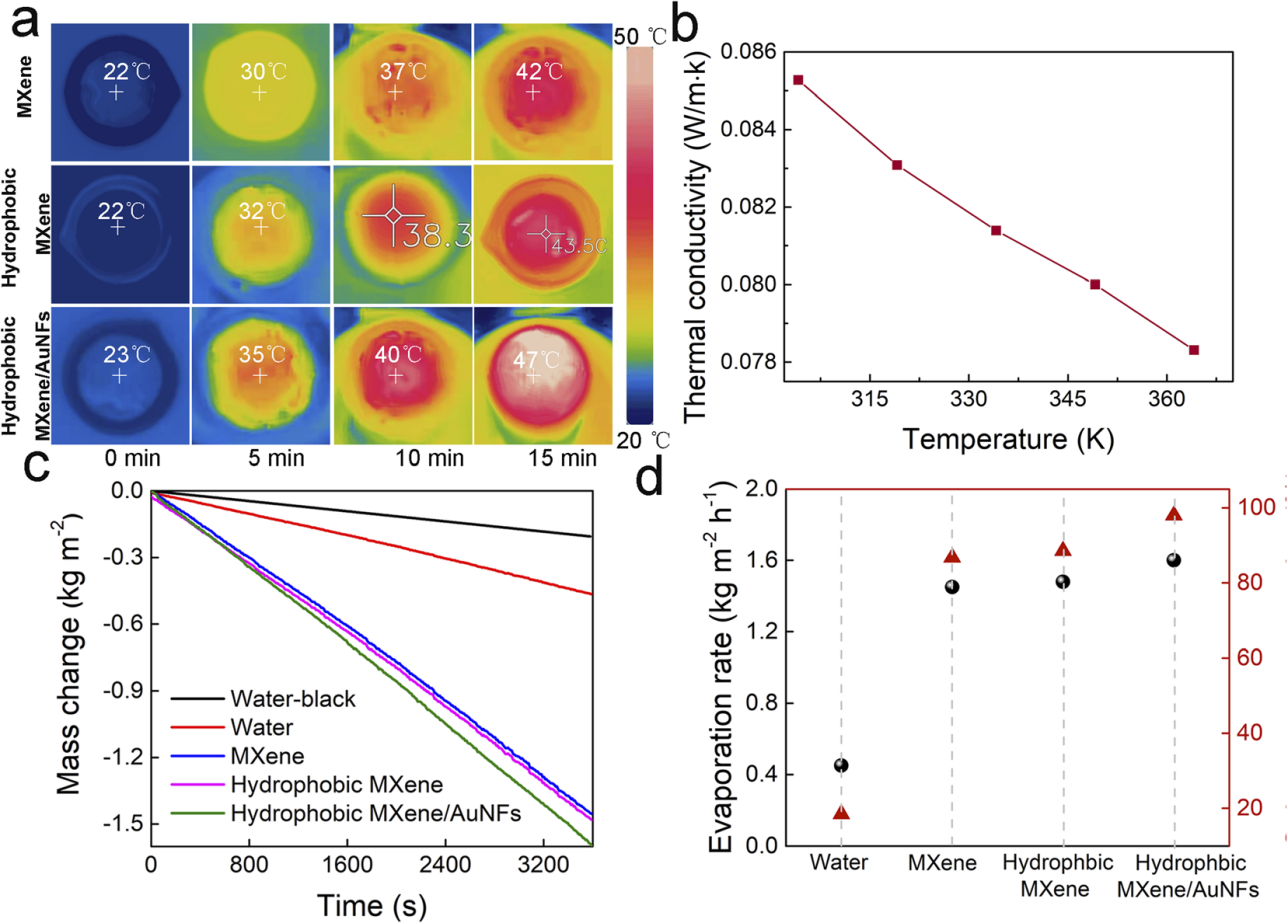
(a) Infrared radiation thermal images of MXene, hydrophobic MXene and hydrophobic MXene/AuNFs membrane under 1.0 kW m^−2^. (b) Thermal conductivity of the hydrophobic MXene/AuNFs membrane at different temperatures. (c) Mass changes of water with different photothermal membranes over time. (d) Evaporation rates and solar-vapor efficiency of water, MXene, hydrophobic MXene and hydrophobic MXene/AuNFs membranes under 1.0 kW m^−2^.

The solar desalination experiment was tested using the evaporation device, as shown in Fig. S3.† The photothermal membranes could self-float upon the water in a glass beaker. A polyethylene foam acts as a thermal isolation device between the photothermal film and the bulk water. Sponge cloth provides a rich water channel due to its excellent wicking effect. Having confirmed the enhanced light absorption of the hydrophobic MXene/AuNFs membrane, the conversion efficiency of light-water evaporation of the hydrophobic MXene/AuNFs were then explored. The thermal localization effect was studied by measuring the surface temperature of photothermal films under one sun with an infrared (IR) camera. As shown in Fig. 4a, the surface temperature of the floating hydrophobic MXene/AuNFs sharply increased from 23 to 47 °C within 15 min which was much higher than that of the MXene (42 °C, Fig. 4a) and hydrophobic MXene (43.5 °C, Fig. 4a). Moreover, the hydrophobic MXene/AuNFs presents low thermal conductivity of 0.08 W mK^−1^ as shown in Fig. 4b, which is conducive to reducing the heat loss into the huge water body by improving the local surface temperature of the membrane. With its high solar absorption ability, low heat loss rate, good hydrophobicity, and high photothermal conversion efficiency, hydrophobic MXene/AuNFs can be used as an efficient light-driven water evaporator in the field of seawater purification.

The light-driven steam generation performance of hydrophobic MXene/AuNFs membrane assembled on our solar desalination device was evaluated under normal solar illumination (1 kW m^−2^). As shown in Fig. S4,† the vapor can be clearly observed in the hydrophobic MXene/AuNFs membrane under simulated solar illumination of 4.0 kW m^−2^, indicating that the water evaporates quickly.

The solar evaporation performance was further demonstrated by measuring the variation curve of water mass with simulation of solar radiation time. As shown in Fig. 4c, the evaporation rates of pure water after solar irradiation is 0.46 kg m^−2^ h^−1^, well the presence of MXene and hydrophobic MXene membranes increase to 1.45 and 1.48 kg m^−2^ h^−1^ respectivly, which are more than three times as much as pure water. Replacing the MXene film with hydrophobic MXene/AuNFs membrane, the evaporation rates is significantly increased, reaching 1.59 kg m^−2^ h^−1^, which is much higer than the MXene and hydrophobic MXene membrane, indicating that AuNFs improves the efficiency of solar water evaporation. The diameter of the photothermal film used in the water evaporation experiment was 4 cm, so the hydrophobic MXene/AuNFs film produces about 2.0 g of water per hour.

Here, the corresponding solar conversion efficiency for evaporation (*η*) of hydrophobic MXene/AuNFs membrane device has been investigated, which is defined as *η* = (Δ*m* × *h*_LV_)/*I*, *h*_LV_ = *H*_sen_ + Δ*H*_vap_. Where Δ*m* is the mass of evaporated water, *H*_sen_ is the sensible heat, Δ*H*_vap_ is latent heat of evaporation and *I* is the light power (1 kW m^−2^). The *η* of hydrophobic MXene/AuNFs membrane device is as high as 97.8% at the light density of 1 kW m^−2^ (Fig. 4d), which is higher than most of interfacial solar steam generation devices based on different kinds of photothermal materials reported by other research groups^51–53^ under 1 kW m^−2^ illumination intensity (Table S1†). The excellent solar thermal efficiency of the hydrophobic MXene/AuNFs based solar water purification device can be attributed to the unique properties of the hydrophobic MXene/AuNFs composite membrane, including perfect light absorption, low thermal conductivity, high water supply efficiency and smooth steam escape. First, the unique three-dimensional multi-branch structure of AuNFs greatly enhances the light absorption and photothermal conversion efficiency of MXene, achieving good photothermal results in solar water purification devices. Second, the low thermal conductivity of hydrophobic MXene/AuNFs makes it an effective thermal barrier, isolating heat from bulk water and setting the stage for efficient solar-powered water evaporation. Third, the honeycomb structure of hydrophobic MXene/AuNFs (Fig. S1†) provides abundant channels for water transport and steam evaporation. Moreover, due to the strong hydrophobicity of fluorosilanes loaded on the surface of MXene/AuNFs, there are fewer salt crystals on the surface of hydrophobic MXene/AuNFs even after continuous evaporation for 15 h under 1 kW m^−2^ solar irradiation.

**Fig. 5:**
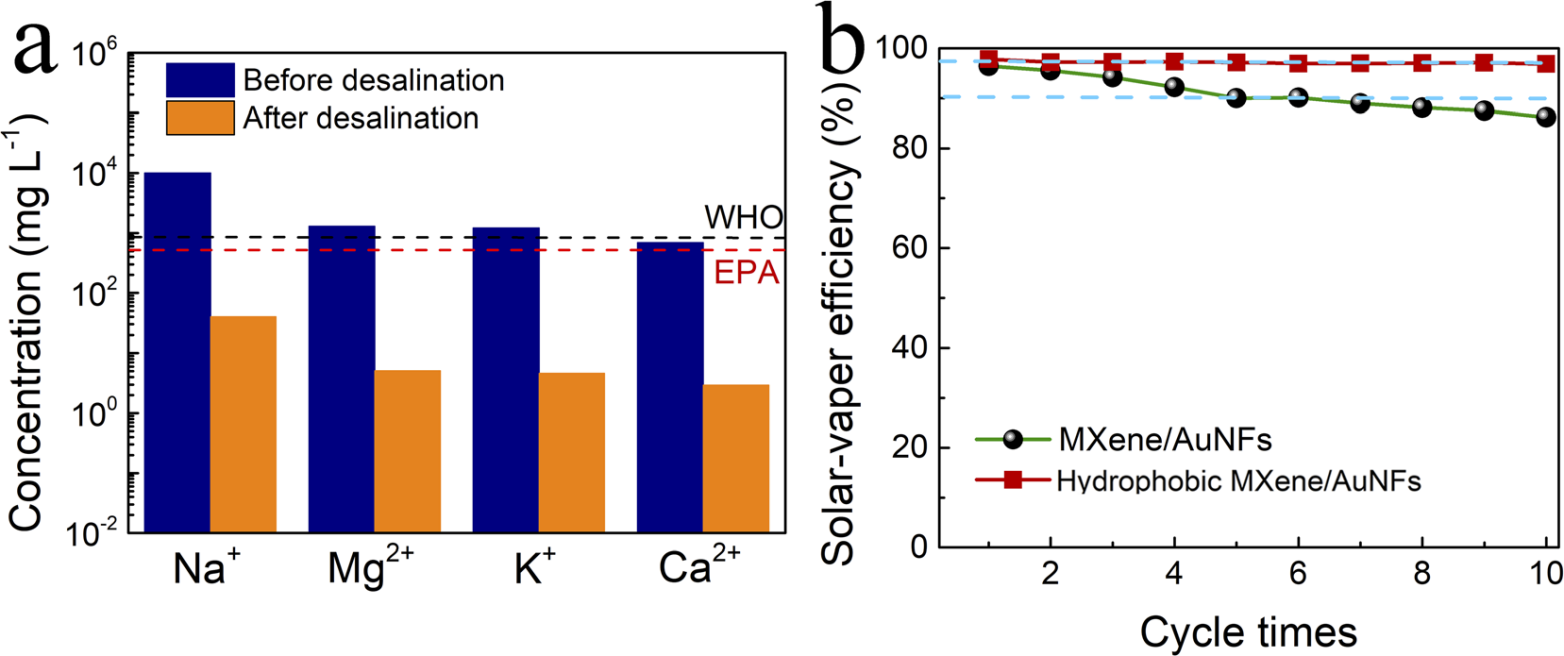
(a) Measured concentrations of four ions in a simulated seawater sample before and after solar desalination by hydrophobic MXene/AuNFs membrane. (b) Cycling performance of MXene/AuNFs and hydrophobic MXene/AuNFs membranes under 1.0 kW m^−2^ for 10 times with 60 minutes per cycle.

In order to verify the practicability of hydrophobic MXene/AuNFs membrane, we designed a seawater desalination device, as shown in Fig. S5,† with a hemispherical condensing chamber covered above the desalination device. We used simulated seawater in our desalination experiments. Under the irradiation of simulated solar light, simulated seawater was continuously transported to the surface of membrane through siphonage, and the vapor generated at the solar water evaporator surface evaporates from the hydrophobic MXene/AuNFs membrane. The water vapor escaping from the surface of the membrane condenses into water droplets in the hemispherical condensing chamber, which slide down the inner wall of the condensing chamber to the bottom and flow into the external container to be collected. The concentrations of four major ions of Na^+^, K^+^, Mg^2+^ and Ca^2+^ are significantly reduced over 99.4% rejection rate compared with the initial simulated seawater after using hydrophobic MXene/AuNFs membrane for solar-driven photothermal desalination (Fig. 5a). The salt content of the water recovered from the installation meets standards set by World Health Organization (WHO) and United States Environmental Protection Agency (EPA).^54^

To verify whether the solar water evaporator has prospects in practical applications, it is also necessary to verify its stability and repeatability. As an efficient and promising solar-driven vapor generation device, it is also necessary to verify its stability and reusability in practical applications. Similarly, the stability and reusability of hydrophobic MXene/AuNFs membrane was tested and characterized by the device shown in Fig. S5.† Under the same conditions, the hydrophobic MXene/AuNFs membrane solar-driven evaporation experiment was tested 10 cycles. For each cycle, the membrane illuminated for 60 minutes by one sun. At the end of each cycle, the wetted hydrophobic MXene/AuNFs film was placed on a paper towel to dry at room temperature for the next test. After cyclic testing, we found that the hydrophobic MXene/AuNFs photothermal film has exceptional stability, even if it is exposed to simulated solar light for a long time, its water evaporation efficiency remains almost constant. As shown in Fig. 5b, after 10 cycles of solar steam generation experiment, the photothermal conversion efficiency of hydrophobic MXene/AuNFs membrane remains at 99.3%, far exceeding 82.1% for the efficiency of MXene/AuNFs film. Due to the non-wettable surface of the MXene/AuNFs repels water naturally, which also prevent salt accumulation on the membrane surface. The hydrophobic MXene/AuNFs membrane acts as a solute blocker to ensure evaporation efficiency and stability during solar desalination. The above results prove that hydrophobic MXene/AuNFs nanocomposite has great potential in the field of solar-powered vapor generation.

## Conclusions

In conclusion, we have successfully developed a promising photothermal material hydrophobic MXene/AuNFs, which can be used as versatile nanoarchitectonics for interfacial solar steam devices. In this paper, AuNFs *in situ* decoration on MXene surface was studied for the first time, and the prepared MXene/AuNFs composite showed good photothermal conversion efficiency. At the same time, the surface of MXene/AuNFs composite was modified with fluorosilane to change its hydrophobicity. Due to the three-dimensional multi-branch structure inherited from AuNFs and the excellent photothermal conversion ability of MXene, hydrophobic hydrophobic MXene/AuNFs membrane has a wide light absorption capacity, which greatly improves the solar energy utilization rate. In addition, the hydrophobic surface modified by fluorosilane makes the MXene composite photothermal membrane more stable and can prevent salt crystallization on the surface. Outstanding characteristics such as high light absorption, efficient photothermal conversion capability and salt resistance of hydrophobic surface make our steam generation device exhibit excellent solar evaporation performance and extraordinary stability. This design has an extremely high conversion efficiency of light to heat up to 97.8% and a water evaporation rate of 1.59 kg m^−2^ h^−1^ under one sun, which is comparable to the most advanced solar-driven water evaporator. Hydrophobic MXene/AuNFs has many advantages, including low cost, simple preparation and recyclability, which provides a new design strategy for rational design and creation of efficient new solar-driven water evaporator.

## Author contributions

Chunjiao Liu: conceptualization, methodology, investigation, visualisation, writing original draft.

## Conflicts of interest

All authors disclosed no relevant relationships.

## Supplementary Material

RA-014-D3RA08157E-s001
