# Supplementary material for: A durable hydrophobic photothermal membrane based on a honeycomb structure MXene for stable and efficient solar desalination
Source: RSC Adv. 2024 Apr 2;14(15):10370–7. doi: 10.1039/d3ra08157e (PMC10985539; doi:10.1039/d3ra08157e)
Supplement: RA-014-D3RA08157E-s001 [file RA-014-D3RA08157E-s001.pdf]

Electronic Supplementary Material (ESI) for RSC Advances.
This journal is © The Royal Society of Chemistry 2024

# Durable hydrophobic photothermal membrane based on honeycomb structure MXene for stable and efficient Solar Desalination.

Chunjiao Liu*

*Institute of Xinyang Vocational and Technical College, Xinyang, Henan 464000, China*

*Corresponding author. E−mail:liuchunjiao_2006@126.com (C. Liu).

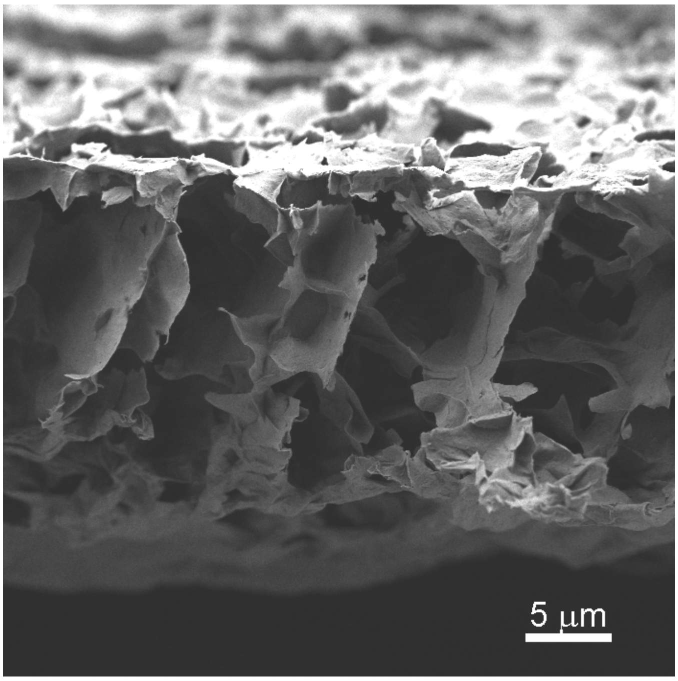


**Fig. S1.** Cross-sectional SEM images of hydrophobic MXene/AuNFs membrane. The thickness is about 18 μm.

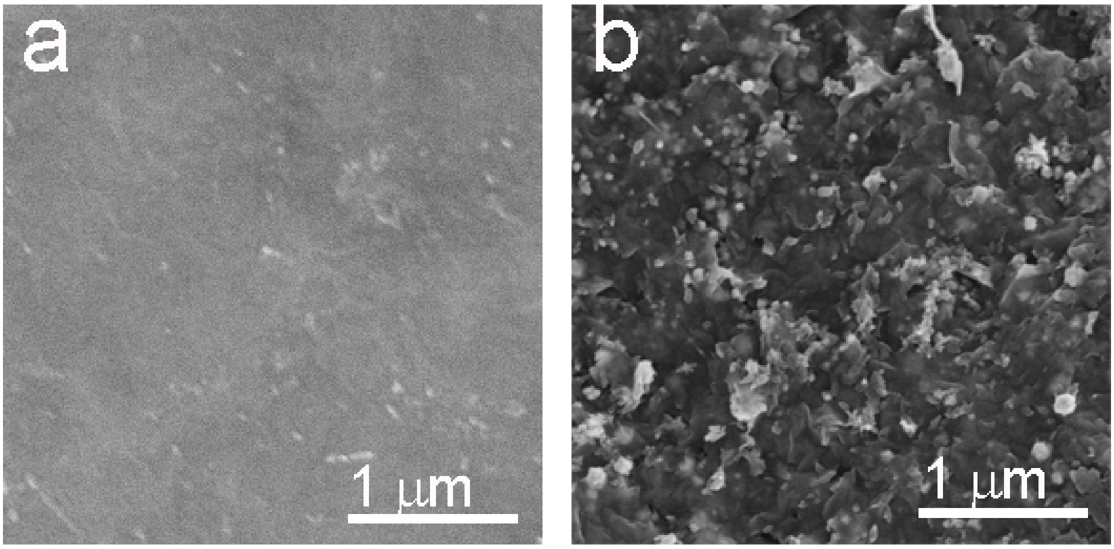


**Fig. S2.** Scanning electron microscopy (SEM) images of pristine MXene (a) and hydrophobic MXene/AuNFs (b).

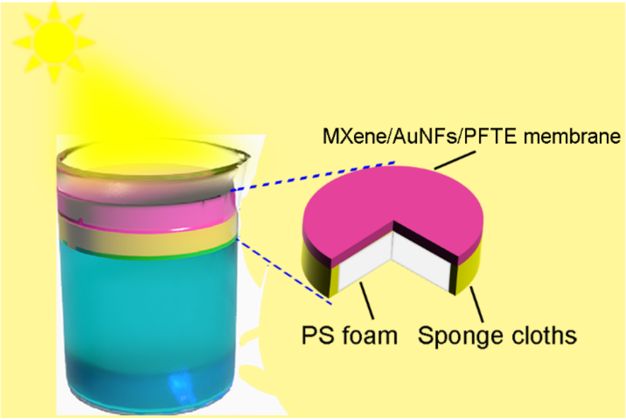


**Fig. S3.** The schematic drawing of solar driven water evaporator.

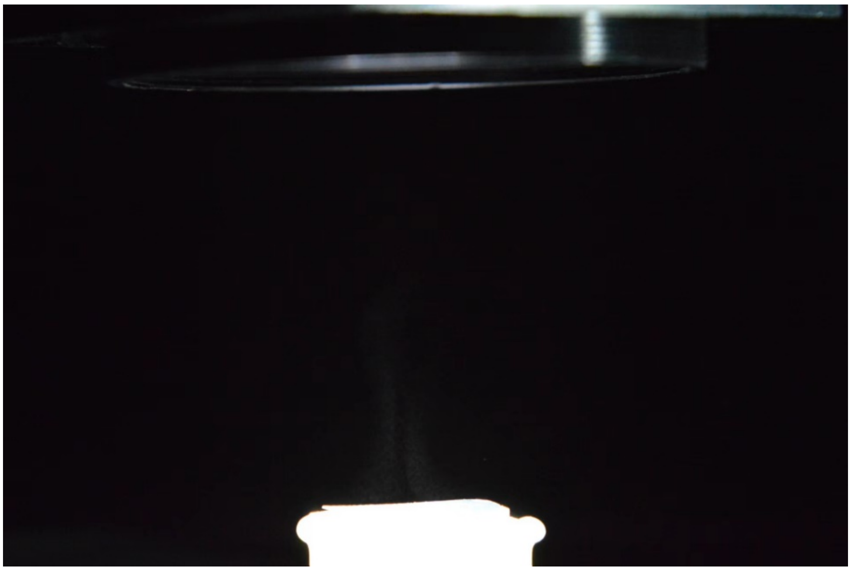

**Fig. S4**. Digital image showing visible steam generation enabled by the hydrophobic MXene/AuNFs membrane under 4 kW $m^{-2}$ illumination.

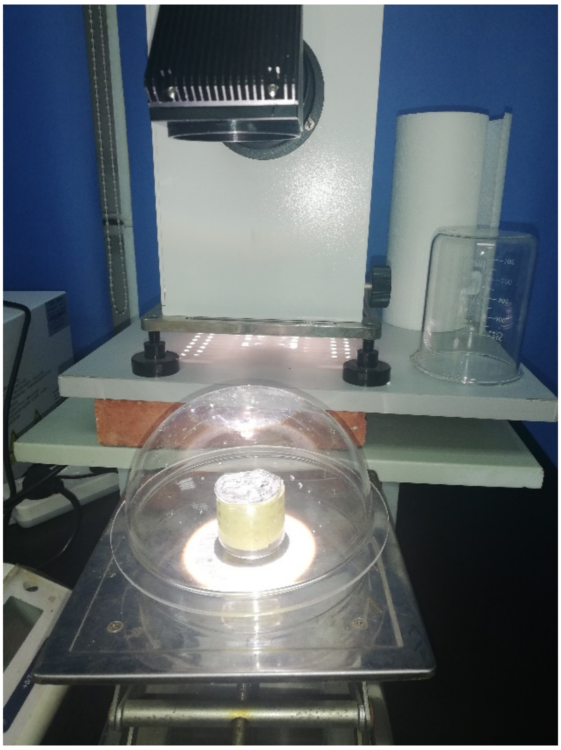

**Fig. S5**. Digital image of the water desalination device for freshwater production.

**Table S1.** A comparison of the solar steem generation performance of our results with some typical published data.

| Photothermal Materials | water evaporation rates (Kg $m^{-2}$ $h^{-1}$) | Efficiencies (%, 1 sun) | Efficiencies (%, 2 sun) | Efficiencies (%, x sun) | Ref. No. |
|---|---|---|---|---|---|
| hydrophobic MXene/AuNFs | 1.59 | 97.8 | □ | □ | This work |
| HPCM/PHS | 1.38 | 90.8 | □ | □ | [1] |
| HN/CNT | 1.09 | 83.2 | □ | 92.8 (10 sun) | [2] |
| In-air calcinated MS | 1.98 | 92 | □ | □ | [3] |
| $Cu_2SnSe_3$ | 1.657 | 86.6 | □ | □ | [4] |
| carbon black/hollow glass microspheres | 1.38 | 82.1 | □ | □ | [5] |
| AuNR/SWNT film | 1.85 | 82 | □ | 94 (5 sun) | [6] |
| p-PEGDA−PANi | 1.4 | 91.5 | □ | □ | [7] |
| CuS/MCE | 1.12 | ~80±2.5 | □ | □ | [8] |
| MXene-PVDF | - | 84 | □ | □ | [9] |
| $WO_{2.9}$ nanorods | 1.28 | 81 | □ | □ | [10] |
| activated carbon fiber | 1.22 | 79.4 | □ | □ | [11] |
| hydrophobic MXene | 1.31 | 71 | □ | □ | [12] |
| N-doped graphene aerogel | 1.558 | 90 | □ | □ | [13] |
| wasted rice straw | 1.2 | 75.8 | □ | □ | [14] |
| PPy-coated PVDF | 1.70 | 93.8 | □ | □ | [15] |
| polymer aerogel | 0.987 | 81 | 85 | 88 (3 sun) | [16] |
| black titania nanocages | 1.13 | 70.9 | □ | □ | [17] |
| PAAm-radial aerogel | 2.0 | 85.7 | □ | □ | [18] |
| PEG $MoS_2$-cotton cloth | 1.287 | 80.5 | 86.8 | 90.3 (5 sun) | [19] |
| GDY-based foam | 1.55 | 91 | □ | □ | [20] |

**References**


[1] X. Ma, W. Fang, Y. Guo, Z. Li, D. Chen, W. Ying, et al., Hierarchical Porous SWCNT Stringed Carbon Polyhedrons and PSS Threaded MOF Bilayer Membrane for Efficient Solar Vapor Generation, Small 15(15) (2019) 1900354.

[2] Z.-C. Xiong, Y.-J. Zhu, D.-D. Qin, F.-F. Chen, R.-L. Yang, Flexible Fire-Resistant

Photothermal Paper Comprising Ultralong Hydroxyapatite Nanowires and Carbon Nanotubes for Solar Energy-Driven Water Purification, Small 14(50) (2018) 1803387.
[3] F. Gong, H. Li, W. Wang, J. Huang, D. Xia, J. Liao, et al., Scalable, eco-friendly and ultrafast solar steam generators based on one-step melamine-derived carbon sponges toward water purification, Nano Energy 58 (2019) 322-330.
[4] Y. Yang, H. Zhao, Z. Yin, J. Zhao, X. Yin, N. Li, et al., A general salt-resistant hydrophilic/hydrophobic nanoporous double layer design for efficient and stable solar water evaporation distillation, Mater Horiz 5(6) (2018) 1143-1150.
[5] W. Zhang, G. Zhang, Q. Ji, H. Liu, R. Liu, J. Qu, Capillary-Flow-Optimized Heat Localization Induced by an Air-Enclosed Three-Dimensional Hierarchical Network for Elevated Solar Evaporation, ACS Appl. Mater. Interfaces 11(10) (2019) 9974-9983.
[6] Y. Yang, X. Yang, L. Fu, M. Zou, A. Cao, Y. Du, et al., Two-Dimensional Flexible Bilayer Janus Membrane for Advanced Photothermal Water Desalination, ACS Energy Lett. 3(5) (2018) 1165-1171.
[7] X. Yin, Y. Zhang, Q. Guo, X. Cai, J. Xiao, Z. Ding, et al., Macroporous Double-Network Hydrogel for High-Efficiency Solar Steam Generation Under 1 sun Illumination, ACS Appl. Mater. Interfaces 10(13) (2018) 10998-11007.
[8] Z. Guo, X. Ming, G. Wang, B. Hou, X. Liu, T. Mei, et al., Super-hydrophilic copper sulfide films as light absorbers for efficient solar steam generation under one sun illumination, Semicond. Sci. Technol. 33(2) (2018) 025008.
[9] R. Li, L. Zhang, L. Shi, P. Wang, MXene Ti3C2: An Effective 2D Light-to-Heat Conversion Material, ACS Nano 11(4) (2017) 3752-3759.

[10] L. Sun, Z. Li, R. Su, Y. Wang, Z. Li, B. Du, et al., Phase-Transition Induced Conversion into a Photothermal Material: Quasi-Metallic WO2.9 Nanorods for Solar Water Evaporation and Anticancer Photothermal Therapy, Angew. Chem. Int. Ed. 57(33) (2018) 10666-10671.

[11] H. Li, Y. He, Y. Hu, X. Wang, Commercially Available Activated Carbon Fiber Felt Enables Efficient Solar Steam Generation, ACS Appl. Mater. Interfaces 10(11) (2018) 9362-9368.

[12] J. Zhao, Y. Yang, C. Yang, Y. Tian, Y. Han, J. Liu, et al., A hydrophobic surface enabled salt-blocking 2D Ti3C2 MXene membrane for efficient and stable solar desalination, J. Mater. Chem. A 6(33) (2018) 16196-16204.

[13] B. Huo, D. Jiang, X. Cao, H. Liang, Z. Liu, C. Li, et al., N-doped graphene /carbon hybrid aerogels for efficient solar steam generation, Carbon 142 (2019) 13-19.

[14] Q. Fang, T. Li, Z. Chen, H. Lin, P. Wang, F. Liu, Full Biomass-Derived Solar Stills for Robust and Stable Evaporation To Collect Clean Water from Various Water-Bearing Media, ACS Appl. Mater. Interfaces 11(11) (2019) 10672-10679.

[15] Y. Wang, C. Wang, X. Song, M. Huang, S.K. Megarajan, S.F. Shaukat, et al., Improved light-harvesting and thermal management for efficient solar-driven water evaporation using 3D photothermal cones, J. Mater. Chem. A 6(21) (2018) 9874-9881.

[16] P. Mu, W. Bai, Z. Zhang, J. He, H. Sun, Z. Zhu, et al., Robust aerogels based on conjugated microporous polymer nanotubes with exceptional mechanical strength for efficient solar steam generation, J. Mater. Chem. A 6(37) (2018) 18183-18190.

[17] F. Jiang, H. Liu, Y. Li, Y. Kuang, X. Xu, C. Chen, et al., Lightweight, Mesoporous,

and Highly Absorptive All-Nanofiber Aerogel for Efficient Solar Steam Generation, ACS Appl. Mater. Interfaces 10(1) (2018) 1104-1112.

[18] W. Xu, Y. Xing, J. Liu, H. Wu, Y. Cui, D. Li, et al., Efficient Water Transport and Solar Steam Generation via Radially, Hierarchically Structured Aerogels, ACS Nano (13) (2019) 7930-7938.

[19] Z. Guo, G. Wang, X. Ming, T. Mei, J. Wang, J. Li, et al., PEGylated Self-Growth MoS2 on a Cotton Cloth Substrate for High-Efficiency Solar Energy Utilization, ACS Appl. Mater. Interfaces 10(29) (2018) 24583-24589.

[20] X. Gao, H. Ren, J. Zhou, R. Du, C. Yin, R. Liu, et al., Synthesis of Hierarchical Graphdiyne-Based Architecture for Efficient Solar Steam Generation, Chem. Mater. 29(14) (2017) 5777-5781.
